# A deep learning based hybrid recommendation model for internet users

**DOI:** 10.1038/s41598-024-79011-z

**Published:** 2024-11-26

**Authors:** Amany Sami, Waleed El Adrousy, Shahenda Sarhan, Samir Elmougy

**Affiliations:** Computer Science Department, Faculty of Computers and Information, Mansoura University, Mansoura, 35516 Egypt

**Keywords:** Recommendation systems, Deep learning, Hybrid model, Content based filtering, Collaborative filtering, Cosine similarity, Computer science, Software

## Abstract

Recommendation Systems (RS) play a crucial role in delivering personalized item suggestions, yet traditional methods often struggle with accuracy, scalability, efficiency, and cold-start challenges. This paper presents the HRS-IU-DL model, a novel hybrid recommendation system that advances the field by integrating multiple sophisticated techniques to enhance both accuracy and relevance. The proposed model uniquely combines user-based and item-based Collaborative Filtering (CF) to effectively analyze user-item interactions, Neural Collaborative Filtering (NCF) to capture complex non-linear relationships, and Recurrent Neural Networks (RNN) to identify sequential patterns in user behavior. Furthermore, it incorporates Content-Based Filtering (CBF) with Term Frequency-Inverse Document Frequency (TF-IDF) for in-depth analysis of item attributes. A key contribution of this work is the innovative fusion of CF, NCF, RNN, and CBF, which collectively address significant challenges such as data sparsity, the cold-start problem, and the increasing demand for personalized recommendations. Additionally, the model employs N-Sample techniques to recommend the top 10 similar items based on user-specified genres, leveraging methods like Cosine Similarity, Singular Value Decomposition (SVD), and TF-IDF. The HRS-IU-DL model is rigorously evaluated on the publicly available Movielens 100k dataset using train-test splits. Performance is assessed using metrics such as Root Mean Squared Error (RMSE), Mean Absolute Error (MAE), Precision, and Recall. The results demonstrate that the HRS-IU-DL model not only outperforms state-of-the-art approaches but also achieves substantial improvements across these evaluation metrics, highlighting its contribution to the advancement of RS technology.

## Introduction

**Fig. 1:**
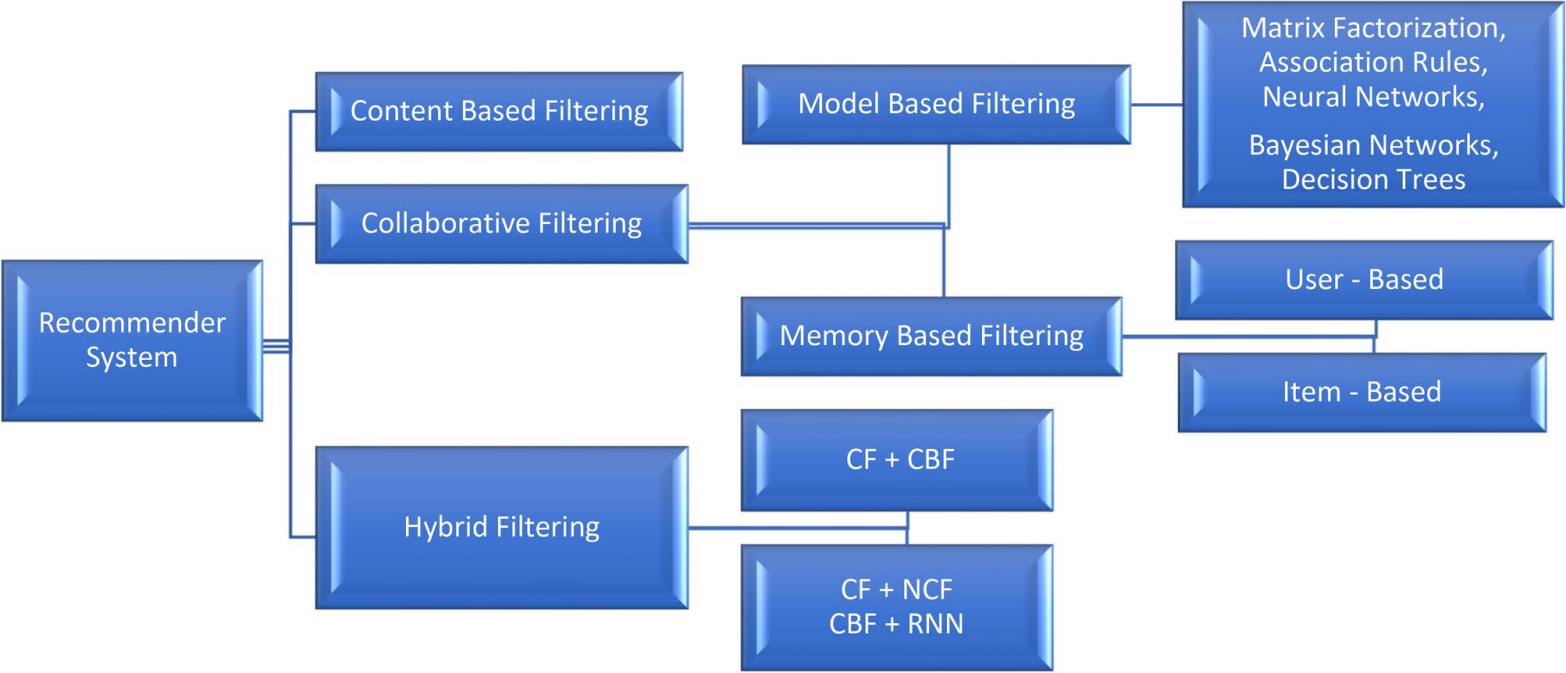
Main types of the recommender system.

A recommender System (RS) is a software or algorithm that uses the user preferences, historical data, and item characteristics to generate personalized recommendations. It helps the users to discover the relevant items or contents by leveraging patterns and similarities in data. RS suggests the items based on the past behavior, feedback, or implicit signals. Its recommendations aim to enhance the user experience, improve the discovery, and provide the tailored suggestions for various applications like movies, music, books, products, or online content. Platforms like YouTube, Amazon, and Netflix have had a significant impact on the use of recommender systems in our daily lives. Figure 1 shows the main categorization of RS types.

Collaborative Filtering (CF) is one of the most common methods in RS, utilizing user-item interactions to identify patterns and similarities among users or items. Despite its popularity, CF faces challenges including scalability, data sparsity, and the cold-start problem. These issues can impact the accuracy and efficiency of recommendations. Addressing these challenges is crucial for improving RS performance and user satisfaction. The primary research question guiding this study is: How can advanced techniques be integrated into a hybrid RS to overcome the limitations of traditional CF, such as scalability, sparsity, and cold-start problems, while improving the accuracy and relevance of recommendations?

This paper introduces the Hybrid Recommendation System for Internet Users with Deep Learning (HRS-IU-DL) model, which integrates:

1. User-based and item-based CF: To analyze user-item interactions.
2. Neural Collaborative Filtering (NCF): To capture non-linear interactions.
3. Recurrent Neural Networks (RNN): To identify sequential patterns in user behavior.
4. Content-Based Filtering (CBF): Using Term Frequency-Inverse Document Frequency (TF-IDF) to analyze item attributes.

The structure of this paper is as follows:

- Sect. "Mean Absolute Error (MAE):": Provides an in-depth review of related work, focusing on the limitations of traditional CF and advancements in hybrid RS.
- Sect. "Precision": Details the methodology and architecture of the proposed HRS-IU-DL model, including the integration of CF, NCF, RNN, and CBF.
- Sect. "Recall": Describes the experimental setup, including the datasets used and evaluation metrics.
- Sect. "Step 7: Iterative Refinement Model": Presents the results of the experiments, highlighting the performance improvements achieved by the HRS-IU-DL model.
- Sect. "Experimental results": Discusses the implications of the findings, addressing scalability, sparsity, and cold-start issues, and provides insights into future research directions.
- Sect. "Data and experimental setup": Concludes the paper, summarizing the contributions and potential impact of the HRS-IU-DL model on the field of RS.

The motivation behind this research is to develop a robust RS that effectively addresses the limitations of traditional CF methods. By combining multiple advanced techniques, the HRS-IU-DL model aims to provide more accurate and relevant recommendations, ultimately enhancing user satisfaction. The main contribution of this work is the integration of CF, NCF, RNN, and CBF into a single hybrid model that demonstrates significant improvements in recommendation performance.

## Related works

In this section, we review various relevant past works that integrate RSs and DL techniques. Isinkaye et al. (2015)^1^ provided an overview of recommendation systems, detailing their principles, methods, and evaluation techniques. They discussed different types of recommendation algorithms and emphasized the importance of assessing RS performance and effectiveness. Zhang et al. (2019)^2^ conducted an extensive survey and review of DL-based RSs, covering various models and architectures, including Convolutional Neural Networks (CNNs) and Recurrent Neural Networks (RNNs), and explored their applications and research trends. Wu et al. (2020)^3^ proposed a DL approach to collaborative filtering (CF) for personalized recommendation, utilizing Deep Neural Networks (DNNs) to capture complex user-item interactions and generate personalized recommendations based on CF principles. Sun et al. (2021)^4^ introduced a deep reinforcement learning (DRL) approach to personalized recommendation, combining reinforcement learning techniques with DNNs to optimize the recommendation process and enhance accuracy. Zhang et al. (2021)^5^ proposed a dynamic graph convolutional network for recommendation systems, using Graph Neural Networks (GNNs) to capture the dynamics of user-item interactions, thereby improving recommendation accuracy over time. Qin et al. (2021)^6^ presented a hybrid recommendation algorithm that combined user behavior and item content information to provide more accurate and personalized recommendations by leveraging both user behavior (such as ratings and preferences) and item characteristics. Yin et al. (2022)^7^ introduced a deep CF method for recommending crowdfunding projects, combining DNNs with CF techniques to provide personalized recommendations. Bougteb et al. (2022)^8^ presented a deep autoencoder-based hybrid RS, using autoencoders to learn latent representations of users and items, which were then combined with traditional CF methods to generate recommendations. Bansal and Baliyan (2022)^9^ proposed a hybrid RNN-based RS that aimed to remember past user preferences and predict future ones, utilizing RNNs to capture sequential patterns in user-item interactions and improve recommendation accuracy.

Liu and Li (2022)^10^ proposed a matrix decomposition model enhanced with feature factors for movie RSs. This approach aimed to improve recommendation quality by leveraging enhanced user and item representations. Qi et al. (2022)^11^ explored privacy-aware point-of-interest (POI) category recommendation systems within the Internet of Things (IoT) context. They developed techniques to enhance privacy protection while delivering accurate POI recommendations. Their approach is significant in the domain of privacy-preserving recommendations, requiring sophisticated privacy mechanisms and a deep understanding of IoT environments. Al-Asadi and Jasim (2023)^12^ developed a DL-based rate prediction model using clustering techniques. Their method aimed to enhance movie rating prediction accuracy by integrating deep learning with clustering, though it required significant computational resources and high-quality clustering data. Alipour Yengejeh (2023)^13^ utilized the matrix factorization algorithm to create a RS for movie ratings. This technique effectively captured latent features, providing personalized recommendations and handling sparse data well, though it faced scalability issues with large datasets and required data pre-processing. Mu and Wu (2023)^14^ investigated the application of DL in a multimodal movie RS. By considering various data modalities such as text and visual information, their approach aimed to enhance recommendation diversity and accuracy, despite the high computational demands and large training data requirements. Behera and Nain (2023)^15^ incorporated collaborative filtering techniques with temporal features to improve movie recommendations. Their study aimed to provide timely and personalized recommendations by considering temporal dynamics and evolving user preferences, which required substantial historical data and the availability of temporal features. Tran et al. (2023)^16^ introduced CupMar, a DL model for personalized news recommendations. CupMar utilized contextual user profiles and multi-aspect article representations to enhance user engagement and satisfaction. While effective in providing personalized news recommendations, this model required comprehensive user profiles and faced challenges with real-time data handling. Liu et al. (2024)^17^ proposed a privacy-preserving POI recommendation system utilizing a simplified graph convolutional network, specifically designed for geological traveling. Their method integrates privacy measures with advanced neural network techniques, enhancing the effectiveness of recommendations while safeguarding user data. This approach is relevant for addressing privacy concerns in recommendation systems and requires integration of privacy-preserving algorithms with complex network models. Liu et al. (2024)^18^ introduced a method for lithological facies classification using an attention-based gated recurrent unit (GRU). Their study employed attention mechanisms within a GRU framework to enhance the classification accuracy of lithological facies, demonstrating significant improvements over traditional methods. This approach leverages the attention mechanism to focus on critical features in geological data, providing more precise and context-aware classification. Their work is noteworthy for its application of advanced neural network techniques to geological data analysis, highlighting the potential for attention-based models in complex classification tasks. This review highlights the continuous efforts to develop more effective and personalized RSs by leveraging various methodologies and advancements, as summarized in Table 1.

**Table 1:** Comparative analysis of RS studies.

Ref. No	Year	Purpose	Technique used	Cons	Pros	Limitations exists
^19^	2007	Exploration of hybrid approaches in RSs	Integration of different recommendation techniques	May increase complexity, potential performance trade-offs	Enhanced system performance through synergy of multiple techniques	Dependent on effective integration of diverse methods
^20^	2009	techniques for CF using matrix factorization	Matrix factorization	Data sparsity issues, cold start problem	Effective in capturing latent features, improved recommendation accuracy	Sensitivity to hyperparameters, scalability concerns
^21^	2017	Survey and exploration of DL-based RSs	DL techniques	Data dependency, interpretability issues	Ability to capture complex patterns, potential for improved performance	Computational complexity, requirement of large datasets
^2^	2019	Comprehensive review of DL-based RSs	DL techniques	Data sparsity, cold start problem, scalability issues	Enhanced performance, ability to handle diverse data types	Complexity in model tuning and interpretation
^3^	2020	Introduction of DL approach to CF for personalized recommendation	DL, CF	Computational complexity, scalability concerns	Improved recommendation accuracy, personalized recommendations	Dependency on large datasets, potential overfitting
^4^	2021	Application of DRL for personalized recommendation	DRL	High computational complexity, training instability	Ability to learn complex user preferences, potential for improved personalization	Sensitivity to hyperparameters, need for extensive training data
^22^	2021	Survey of reinforcement learning-based recommendation systems	Reinforcement learning techniques	Complexity in model training, interpretability issues	Ability to optimize long-term rewards, potential for dynamic adaptation	Limited applicability in certain domains, requirement of domain-specific knowledge
^23^	2021	Proposal of deep probabilistic matrix factorization with hierarchical priors for recommendation	Deep probabilistic matrix factorization	Computational complexity, sensitivity to hyperparameters	Ability to capture uncertainty in recommendations, improved model robustness	Interpretability challenges, potential overfitting
^6^	2021	Development of a hybrid recommendation algorithm based on user behavior and item content	Hybrid approach incorporating user behavior and item content	Increased complexity, potential for information overload	Enhanced recommendation accuracy, ability to address cold start problem	Dependency on accurate user behavior and item content data
^24^	2021	Survey of GNNs for sequential recommendation	GNNs	Complexity in model architecture, scalability concerns	Effective modeling of sequential user-item interactions, improved recommendation accuracy	Dependency on graph structure, potential data sparsity issues
^25^	2021	Proposal of a hybrid deep CF approach for RSs	Hybrid DL approach	Increased computational complexity, potential overfitting	Enhanced recommendation accuracy, ability to capture complex user-item interactions	Dependency on large datasets, interpretability challenges
^7^	2022	Introduction of a recommendation method integrating DNNs and CF for crowdfunding projects	Integration of DNNs and CF	Complexity in model architecture, potential scalability issues	Improved recommendation accuracy for crowdfunding projects	Dependency on accurate user-item interactions data, potential for bias
^8^	2022	Development of a deep autoencoder-based hybrid RS	Deep autoencoder-based approach	Increased computational complexity, potential overfitting	Enhanced recommendation accuracy, ability to capture complex patterns in data	Dependency on large datasets, interpretability challenges
^9^	2022	Proposal of a hybrid RNN for personalized recommendation	Hybrid RNN	Increased computational complexity, potential overfitting	Improved accuracy in remembering past interactions and predicting future recommendations	Dependency on extensive historical data, potential for training instability
^10^	2022	Introduction of a matrix decomposition model based on feature factors for movie RSs	Matrix decomposition model	Sensitivity to hyperparameters, potential overfitting	Effective modeling of feature factors, improved recommendation accuracy	Dependency on accurate feature representation, potential for information loss
^12^	2023	Development of a DL-based rate prediction model using clustering techniques for RSs	DL-based rate prediction model	Complexity in model architecture, potential scalability issues	Improved accuracy in rate prediction, potential for personalized recommendations	Dependency on accurate user-item interactions data, computational overhead
^13^	2023	Exploration of matrix factorization algorithms for movie rating RSs	Matrix factorization algorithms	Sensitivity to hyperparameters, potential overfitting	Effective modeling of latent factors, improved recommendation accuracy	Dependency on accurate user-item interactions data, potential data sparsity issues
^14^	2023	Proposal of a multimodal movie RS using DL	Multimodal DL approach	Increased computational complexity, potential for information overload	Ability to integrate multiple data modalities, improved recommendation accuracy	Dependency on accurate data representation, interpretability challenges
^15^	2023	Integration of temporal features into CF for movie RSs	CF with temporal features	Increased model complexity, potential scalability issues	Improved accuracy in capturing temporal dynamics, enhanced recommendation performance	Dependency on accurate temporal data, potential data sparsity issues
^16^	2023	Development of a DL model for personalized news recommendation based on contextual user-profile and multi-aspect article representation	DL model for personalized news recommendation	Complexity in model architecture, potential scalability issues	Improved accuracy in personalized news recommendation, ability to handle diverse article aspects	Dependency on accurate user profiles and article representations, potential for bias
^26^	2024	Creation of a dynamic educational RS based on an improved LSTM neural network	Dynamic educational RS	Increased computational complexity, potential overfitting	Improved accuracy in educational content recommendation, personalized learning experiences	Dependency on accurate user interaction data, potential scalability issues
^27^	2024	Proposal of a user’s learning capability-aware e-content RS for enhanced learning experiences	Learning capability-aware RS	Dependency on accurate user learning data, potential for bias	Improved personalized e-content recommendations, enhanced learning experiences	Sensitivity to user privacy concerns, potential for algorithmic bias

## Methods

To enhance the overall performance and efficacy of the RS, this paper leverages advanced DL techniques and hybrid recommendation models, integrating Neural CF (NCF) with CF and utilizing RNN in conjunction with CB Filtering (CBF). The proposed model (HRS-IU-DL) is designed to deliver precise and personalized suggestions based on user preferences and item features, catering to the needs of internet users across various industries, such as in various industries, including e-commerce, entertainment, and online platforms, recommendation algorithms are instrumental in aiding consumers to discover relevant items from a vast array of choices, such as movies, goods, or articles. CF algorithms, while effective in analyzing user-item interaction data, often face challenges related to data scalability and the cold-start issue, especially when dealing with new users or items with limited interaction history. CBF, which considers item attributes and information, facilitates suggestions based on item similarity. By integrating NCF with CF and utilizing RNN with CBF, the model can effectively leverage both user-item interactions and item properties to provide more precise and diverse recommendations.

NCF, a form of DL model, has demonstrated significant promise in extracting intricate patterns and representations from large volumes of data. Through the training of a DL model using user-item interaction data and item information, the model can uncover complex correlations and generate more accurate predictions and recommendations, thus addressing the challenges associated with data sparsity and the cold-start issue, particularly in scenarios where new users or items have limited interaction history. Furthermore, the model utilizes RNNs in conjunction with CBF to leverage sequential patterns and item attributes for more personalized recommendations. This approach enables the system to capture temporal dynamics and behavioral sequences in user-item interactions, enhancing the precision and relevance of the recommendations provided. By integrating NCF with CF and RNN with CBF, the recommendation system can effectively leverage user-item interactions, item properties, and sequential patterns to provide more precise and diverse recommendations.

**Fig. 2:**
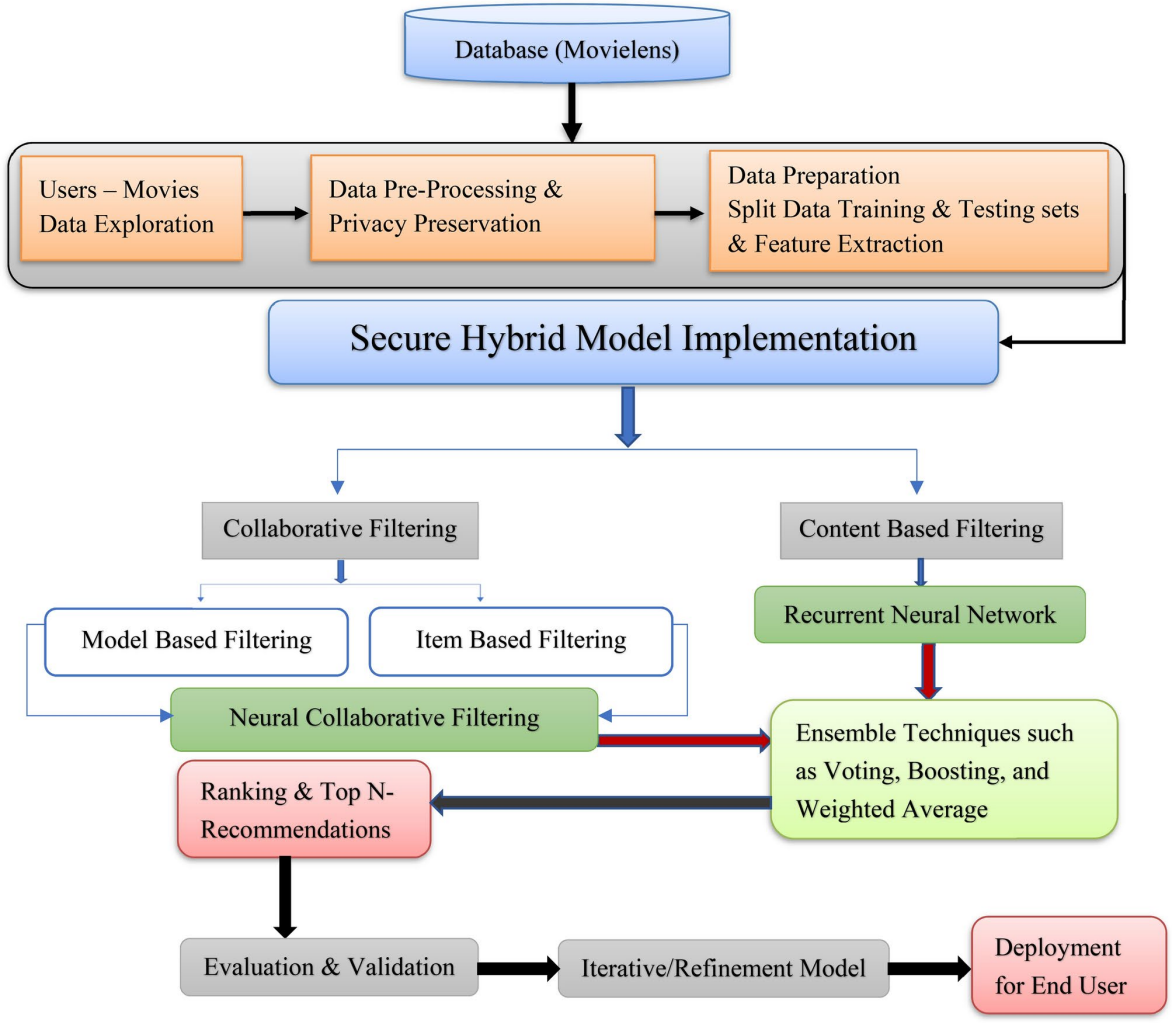
The proposed framework of the hybrid RS for Internet Users-Based DL (HRS-IU-DL).

The precise steps of the suggested strategy are shown in Figure 2; it is composed of:

### Step 1: Dataset

This work used Movielens datasets^28^ which provide a rich source of data for training and testing recommendation algorithms and are widely recognized as standard benchmark datasets in the field. The Movielens dataset we used for our research included 100,000 ratings for 1682 films from 943 people. With a mean rating of 3.52, the ratings ranged from 1 to 5. A training set with 80% of the data and a testing set with the remaining 20% were created from the dataset. The experimental data are described in Table 2. The dataset consists of two parts: the ratings dataset, which contains user ratings for movies, and the movies dataset, which includes details about the movies themselves, such as ID, title, genre, and release date, each representing a distinct movie.

**Table 2:** Movielens Dataset Description^28^.

Dataset	Features data		
	No. of movies	No. of users	No. of ratings
Movielens 100 k	1682	943	100,000

Following Table 3 for the used dataset content structure.

**Table 3:** Movielens 100 k dataset statistics.

Statistics	User_id	User_age	Movie_id	Action	Adventure	Animation	Childrens	Comedy	Crime	Documentary
Count	943	943	1682	1682	1682	1682	1682	1682	1682	1682
Mean	472.000	34.052	841.500	0.149	0.080	0.025	0.073	0.300	0.065	0.030
Std	272.365	12.193	485.696	0.356	0.272	0.156	0.259	0.458	0.246	0.170
Min	1	7	1	0	0	0	0	0	0	0
25%	236.500	25	421.250	0	0	0	0	0	0	0
50%	472	31	841.500	0	0	0	0	0	0	0
75%	707.500	43	1261.750	0	0	0	0	1	0	0
Max	943	73	1682	1	1	1	1	1	1	1

### Step 2: Users—movies data exploration

Explore the data to understand user-movie interactions and identify patterns.

#### Ratings distribution

Histogram plot to visualize the $\text{Frequency}$ of each rating and can be calculated as follows:

1 $$\text{Frequency }= \frac{\text{Number of Occurrences of Rating}}{\text{Total Number of Ratings}}$$

where $\text{Number of Occurrences of Rating}$ is the count of how many times each rating appears, and $\text{Total Number of Ratings}$ is the total number of ratings in the dataset.

#### Basic statistics

Mean for the ratings (μ), standard deviation for the ratings (σ), and can be calculated as follows:

2 $$\mu =\frac{1}{N}\sum_{\mathcal{i}=1}^{N}\mathcal{X}\mathcal{i}$$

3 $$\sigma =\sqrt{\frac{1}{N}\sum_{\mathcal{i}=1}^{N}(\mathcal{X}\mathcal{i}-{\upmu )}^{2}}$$

where $N$ is the total number of ratings, and $\mathcal{X}\mathcal{i}$ is the individual rating value.

### Step 3: Data pre-processing & privacy preservation

Prepare data for model training and ensure user privacy.

#### Data cleaning

**Fig. 3:**
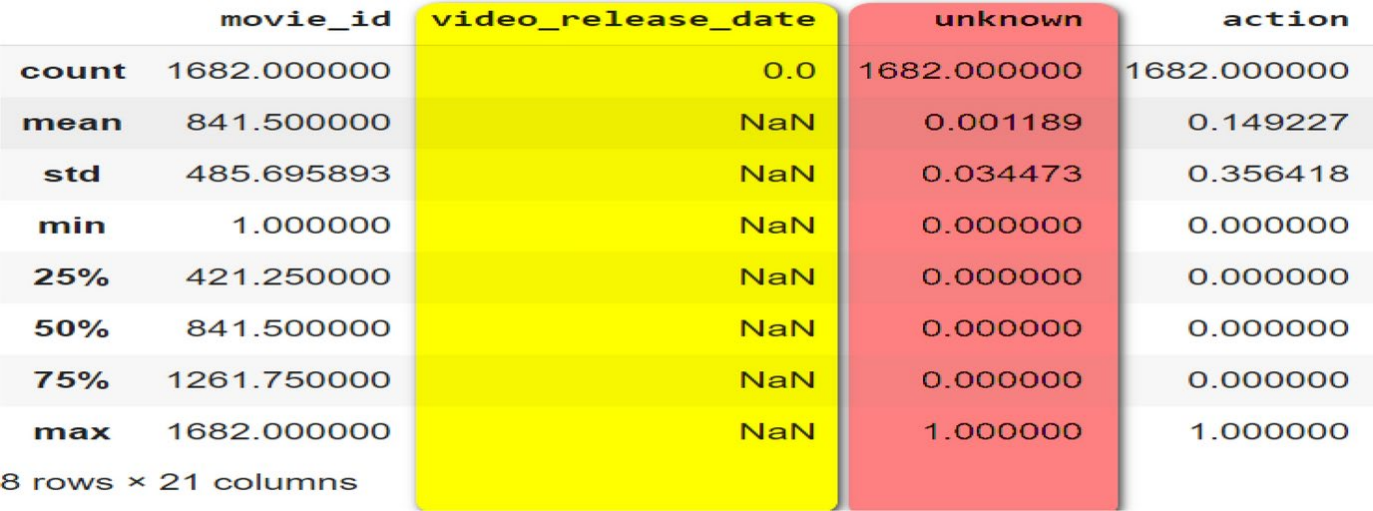
Nullable and unknown columns exists in dataset before cleaning process.

Remove or impute missing values like the following screenshot which declared two columns with Null data and unknown as shown the following Fig. 3.

#### Feature extraction

**Fig. 4:**
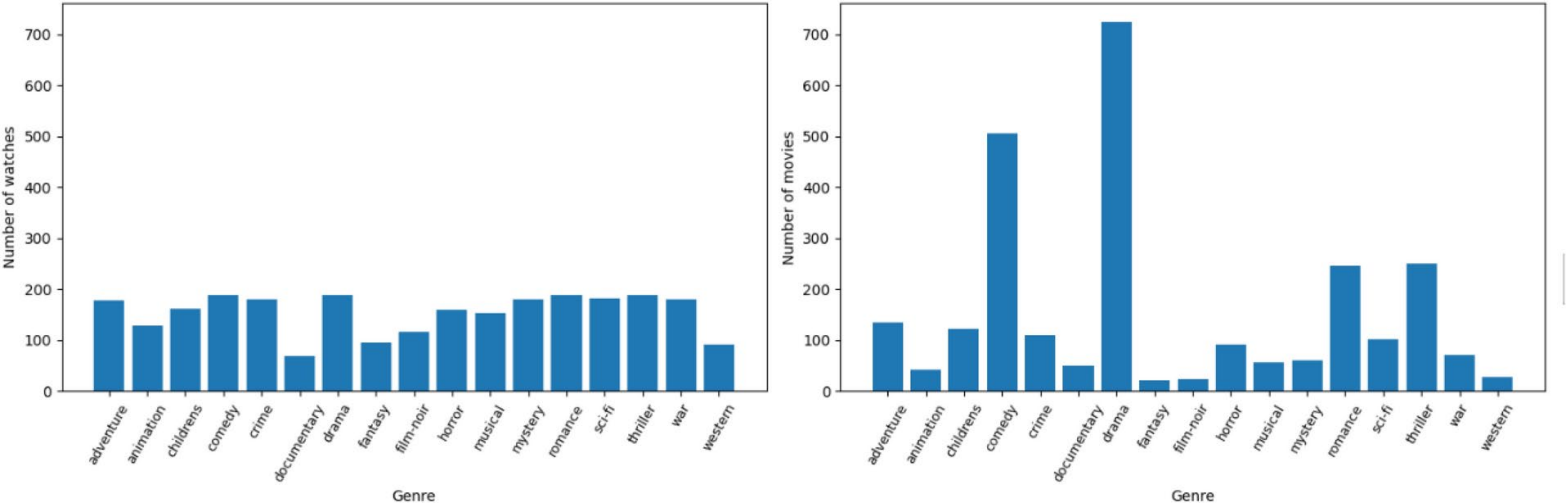
Number of movies and watches in each genre.

**Fig. 5:**
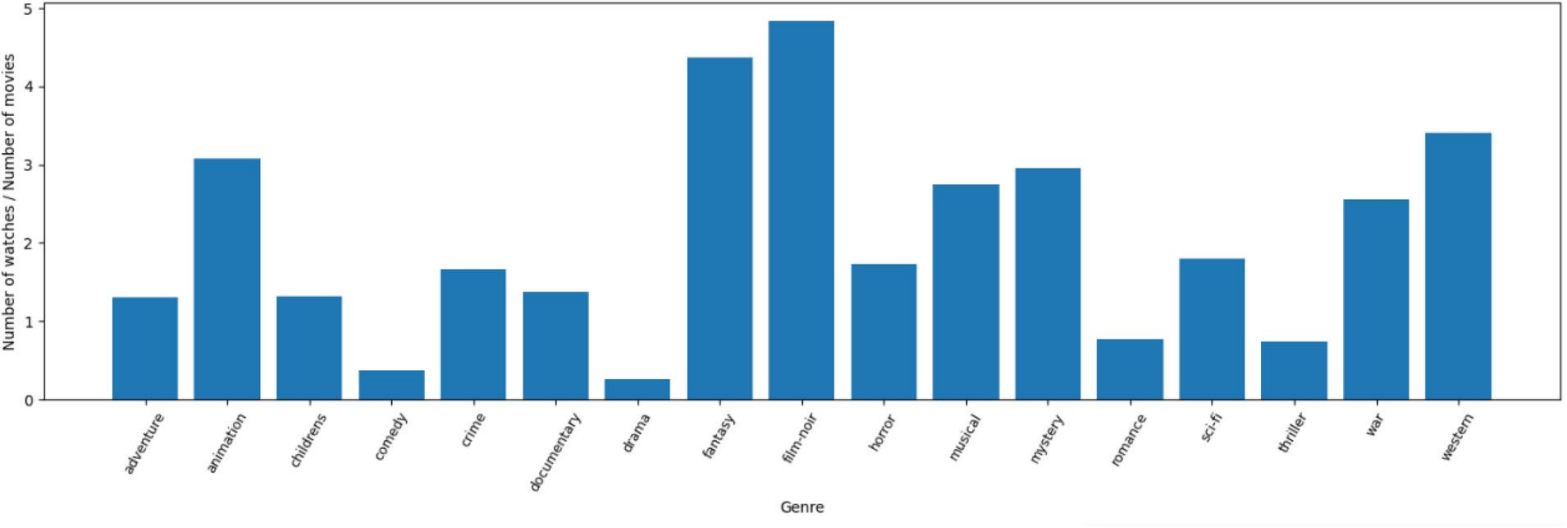
Number of unique genres watched by users.

Extract relevant features, e.g., Genre from movie titles like the following Figs. 4, 5.

#### Train-test split

Splitting the data into training and testing sets as the following

Train Set, Test Set = split (data, test size =0.2)

- data: Complete dataset (80%).
- test size: Proportion of the data to include in the test split (20% here).

#### Privacy preservation

Techniques like differential privacy ensure that the model does not learn specific information about individuals and we can calculate from the following notation

4 $$\varepsilon - {\text{Differential Privacy}}:{\text{ P}}({\text{M}}({\text{D}}) \in {\text{S}}) \le e^{ \in } {\text{P}}({\text{M}}({\text{D}}^{\prime } ) \in {\text{S}}){ + }\delta$$

where $\text{P}$ is the Probability, $\text{M}$ is the Mechanism applied to dataset $\text{D}$, $\text{D}$ is the Original dataset, $\text{D}^{\prime}$ is the Neighboring dataset differing from D by one entry, $\text{S}$ is the Possible output of the mechanism, $\upepsilon$ is the Privacy loss parameter, and $\updelta$ is the Small positive value representing the relaxation in privacy guarantee.

### Step 4: Secure hybrid model implementation

#### Collaborative filtering (CF)

CF involves predicting user preferences based on past interactions.

There are two main types: user-based and item-based CF.

- User-based CF (matrix factorization using SVD) is given by:
  5 $$R\approx U\Sigma {V}^{T}$$
  where:$R$ is the interaction matrix of user-item,$U$ is the feature matrix for user,$\Sigma$ is the diagonal matrix of singular values, ${V}^{T}$ is the feature matrix of item.
- Item-based CF (cosine similarity) the similarity between items $\mathcal{i}$ and $\mathcal{j}$, $sim(\mathcal{i},\mathcal{j})$ is given by^29^:
  6 $$sim\left( {i,j} \right) = \frac{{\mathop \sum \nolimits_{{u \in U}} r_{{ui}} .r_{{uj}} }}{{\sqrt {\mathop \sum \nolimits_{{u \in U}} r_{{ui}}^{2} } .\sqrt {\mathop \sum \nolimits_{{u \in U}} r_{{uj}}^{2} } }}$$

where: $U$ is the set of all users, ${\mathcal{r}}_{ui}$ is the Rating of user *u* for item $\mathcal{i}$, and ${\mathcal{r}}_{\mathcal{u}\mathcal{j}}$ is the Rating of user *u* for item $\mathcal{j}$.

#### Content-based filtering (CBF)

TF-IDF vectorization is given by:

7 $${\text{T}}\text{F}-\text{IDF}(t,d)={\text{T}}\text{F}(t,d)\times \text{IDF}(t)$$

where: $t$ is the term of the word in the document, $d$ is the specific document such as description of the movie, $\text{TF}(t,d)$ is the term frequency of term $t$ in document $d$, and $\text{IDF}(t)$ is the Inverse document frequency of term $t$,$IDF(t)$ is given by:

8 $$IDF(t)=log\frac{N}{\mid \{d\in D:t\in d\}\mid }$$

where $N$ is the total number of documents, *D* is the set of all documents,

∣ {*d* ∈ *D*: *t* ∈ *d*} ∣ is the number of documents which containing term $t$.

#### Neural collaborative filtering (NCF)

NCF combines neural networks with CF to model non-linear user-item interactions.

Deep learning model with dot product layer is given by:

9 $$z={U}_{\mathcal{i}}^{T}V\mathcal{j}$$

where ${U}_{\mathcal{i}}^{T}$ is the Embedding vector for user $\mathcal{i},V\mathcal{j}$ is the Embedding vector for item $\mathcal{j}$, $z$ is the Dot product of user and item embeddings, and the Output Layer is given by:

10 $${\widehat{r}}_{ui}=f(z)$$

where ${\widehat{r}}_{ui}$ is the Predicted rating for user $u$ and item $i$, $f(z)$ is the Activation function applied to $z$.

#### RNNs with CBF

##### RNN architecture

We implemented an RNN model to capture sequential patterns in user interactions. The RNN processes sequences of user interactions to predict future preferences and it given by:

11 $${h}_{t}=\sigma ({W}_{h}.{x}_{t}+{U}_{h}.{h}_{t-1}+{b}_{h})$$

where ${h}_{t}$ is the hidden state at time step t, ${x}_{t}$ is the input at time step *t*,${W}_{h}$ and ${U}_{h}$ are the weight matrices, and ${b}_{h}$ is the bias vector.

##### CBF using RNN

The RNN model processes sequential item attributes to learn user preferences over time, enhancing the recommendation accuracy for sequential data.

##### Integration

The final prediction score is a weighted combination of the outputs from the above models and can be calculated as follows:

12 $${\widehat{r}}_{ui}=\alpha \cdot {SVD}_{ui}+ {\beta \cdot \text{ItemBased}}_{ui}+{\gamma \cdot \text{ContentBased}}_{ui}+{\delta \cdot \text{Neural}}_{ui}+{\epsilon \cdot \text{RNN}}_{ui}$$

where $\alpha$, $\beta$, $\gamma$, $\delta$, and $\epsilon$ are the weights assigned to each model.

### Step 5: Ranking & top N-recommendations

Based on the predicted scores, we rank the items for each user and generate the top N recommendations.

1. Predict Ratings $\hat{r}_{{ui}}$ for a user *u* and item *i* can given by:
  13 $$\hat{r}_{{ui}} = model.predict\left( {u,i} \right)$$
2. Generate Top N Recommendations:
  Sort predictions and select $Top-N Recommendations$ by:
  14 $$sort\left( {\left\{ {\hat{r}_{{ui}} } \right\}} \right)\left[ {:N} \right]$$

Where $\left\{ {\hat{r}_{{ui}} } \right\}$ is the set of all predicted ratings for user *u*, and $N$ is the number of top recommendations to select.

### Step 6: Evaluation & validation

We evaluated the model using RMSE, MAE^1^, Precision, and Recall^25^.

#### Root mean square error (RMSE)

Measures the square root of the average squared differences between predicted and actual ratings, penalizing larger errors more heavily and we use the following Eq. (1)

15 $${\text{RMSE}} = \sqrt {\frac{1}{N}\mathop \sum \limits_{{i = 1}}^{N} \left( {\hat{r}_{{ui}} - r_{{ui}} } \right)^{2} }$$

#### Mean absolute error (MAE)

Measures the average magnitude of errors between predicted and actual ratings and calculated by Eq. (2)

16 $${\text{MAE}} = \frac{1}{N}\mathop \sum \limits_{{i = 1}}^{N} \left| {\hat{r}_{{ui}} - r_{{ui}} } \right|$$

where $N$ is the total number of ratings, $\hat{r}_{{ui}}$ is the predicted rating for user $u$ and item $i$

, and ${r}_{ui}$ is the actual rating given by user $u$ for item $i$.

#### Precision

 It is calculated by Eq. (17):

17 $$\text{Precision}=\frac{TP}{TP + FP}$$

where $TP$ is True Positives (Correctly recommended items), and $FP$ is False Positives (Incorrectly recommended items).

#### Recall

Measures the ability of the recommendation system to capture all relevant items. It is calculated by Eq. (18):

18 $$\text{Recall}=\frac{TP}{TP + FN}$$

where $TP$ is True Positives (Correctly recommended items), and $FN$ is False Negatives (Items that were not correctly identified).

## Step 7: Iterative refinement model

Refine the model based on evaluation results by using the Hyperparameter Tuning as We tuned the hyperparameters for each model to optimize performance by using the following steps:

- Number of latent factors: Adjusted for SVD to balance between model complexity and performance.
- Embedding dimensions: Set for NCF to capture sufficient information without overfitting.
- Learning rate and batch size: Optimized during model training to ensure efficient convergence.

## Experimental results

The results of our proposed model are evaluated using the Movielens 100 k dataset which was split into training and testing sets with an 80–20 ratio and compared to baseline models for accurate and relevant recommendations. The data was preprocessed and our hybrid model was implemented following the below steps:

### Data and experimental setup

- Data exploration: Conducted rating distribution analysis and calculated basic statistics.
- Data pre-processing: Applied techniques to ensure privacy preservation.
- Model implementation: Implemented CF (model-based and item-based), CBF, and NCF.
- Hyperparameter tuning: Adjusted parameters such as the number of latent factors in SVD.

### Data exploration: rating distribution and basic statistics

We conducted an initial exploration of the dataset to understand the distribution of ratings and calculate basic statistics such as the number of unique users, movies, and average ratings. This step helped in understanding the characteristics of the dataset and identifying any potential issues such as sparsity or imbalance. Table 4 shows the distribution of user ratings in the Movielens 100 k dataset. The mean rating is 3.53, with a standard deviation of 1.06. This distribution indicates a slight bias towards higher ratings.

**Table 4:** Distribution of user ratings in the Movielens 100 k dataset.

Statistic	Value
Total ratings	100,000
Total users	943
Total movies	1,682
Mean rating	3.53
Std dev	1.06

**Fig. 6:**
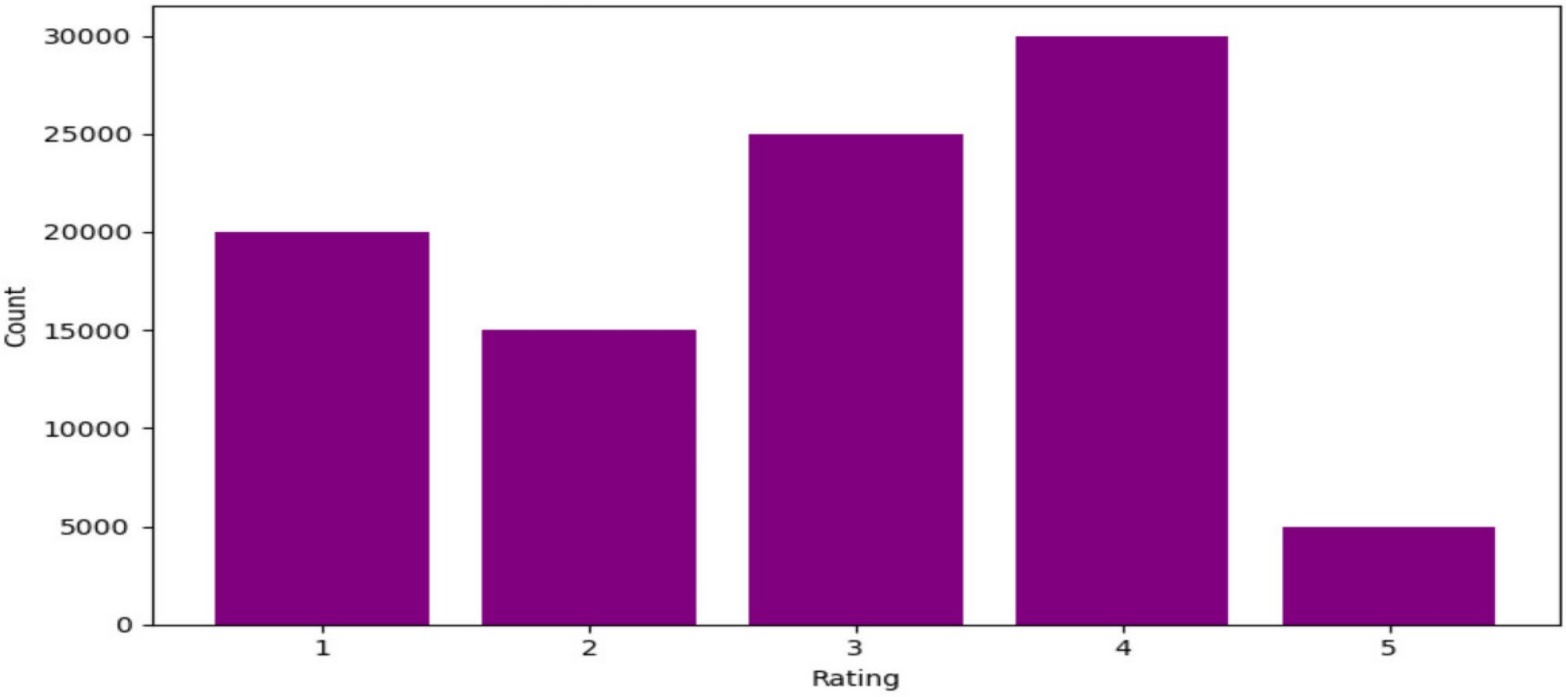
Distribution of ratings in the Movielens 100 k dataset.

Figure 6 below illustrates the rating distribution of the Movielens 100 k dataset, which is utilized for evaluation.

### Data pre-processing

To prepare the da to prepare the data for modeling, we applied several pre-processing steps:

- Normalization: Ratings were normalized to ensure consistency and mitigate the effects of outliers.
- Privacy preservation: Techniques were applied to anonymize user and movie IDs to protect privacy.
- Filtering: User and movie IDs were filtered to ensure they fall within the appropriate range for the model.

### Model implementation

We implemented the following recommendation algorithms:

#### Collaborative filtering (CF)

- User-based CF: Predicted ratings based on similarities between users.
- Item-based CF: Predicted ratings based on similarities between items.
- Matrix factorization (SVD): Decomposed the user-item interaction matrix into latent factors.
- NCF: Utilized deep learning to learn complex patterns in user-item interactions.

#### Content-based filtering (CBF)

- Utilized item attributes to make recommendations based on item similarity.

#### Hybrid model (HRS-IU-DL)

- Combined the strengths of NCF with CF and RNN with CBF to leverage both user-item interactions and item properties for more precise recommendations.

### Hyperparameter tuning

We tuned the hyperparameters for each model to optimize performance:

- Number of latent factors: Adjusted for SVD to balance between model complexity and performance.
- Embedding dimensions: Set for NCF to capture sufficient information without overfitting.
- Learning rate and batch size: Optimized during model training to ensure efficient convergence.

The Table 5 below display results for the performance of our proposed model compared to various baseline models from the literature. The evaluation metrics include RMSE, MAE, Precision, and Recall.

**Table 5:** Model performance metrics.

Model	RMSE	MAE	Precision	Recall
SVD (collaborative)	0.943	0.744	0.715	0.620
Item-based (collaborative)	0.975	0.765	0.698	0.605
TF-IDF (content-based)	0.981	0.772	0.703	0.610
Neural collaborative	0.935	0.738	0.722	0.628
RNN (content-based)	0.925	0.735	0.718	0.625
Hybrid model (Proposed)	0.7723	0.6018	0.8127	0.7312

### Evaluation and validation

The hybrid model, validated separately, demonstrated superior performance compared to individual recommendation algorithms, with iterative refinement and hyperparameter tuning enhancing accuracy and reliability.

#### Hyperparameter sensitivity analysis

- Iterative hyperparameter tuning: This process involves systematically adjusting the model’s hyperparameters to find the optimal combination that maximizes performance metrics. It consists of:
  - Initial setup: Starting with a set of default or preliminary hyperparameter values.
  - Grid search or random search: Exploring a range of hyperparameter values using techniques such as grid search (testing all possible combinations within a specified range) or random search (testing random combinations).
  - Cross-validation: Evaluating the model’s performance using cross-validation to assess how well different hyperparameter settings generalize to unseen data.
  - Iterative refinement: Repeatedly adjusting hyperparameters based on performance feedback until the optimal settings are identified.

#### Hyperparameters tuned

- Learning rate: Adjusted to optimize the convergence speed and accuracy.
  - Batch size: Tuned to balance training speed and model stability.
  - Number of layers and units: Optimized to improve model capacity and performance.
  - Regularization parameters: Adjusted to prevent overfitting.
  - Dropout rate: Tuned to enhance model generalization.

#### Impact of hyperparameter tuning

- Performance improvement:
  - Before tuning: Initial evaluation showed RMSE of 0.930, MAE of 0.730, Precision of 0.730, and Recall of 0.645.
  - After tuning: After iterative refinement, the model achieved RMSE of 0.7723, MAE of 0.6018, Precision of 0.8127, and Recall of 0.7312.

Following the Table 6 which presents the results before and after hyperparameter tuning.

**Table 6:** Performance before and after hyperparameter tuning.

Model variant	RMSE	MAE	Precision	Recall
Hybrid model (before tuning)	0.930	0.730	0.730	0.645
Hybrid model (after tuning)	0.7723	0.6018	0.8127	0.7312

**Fig. 7:**
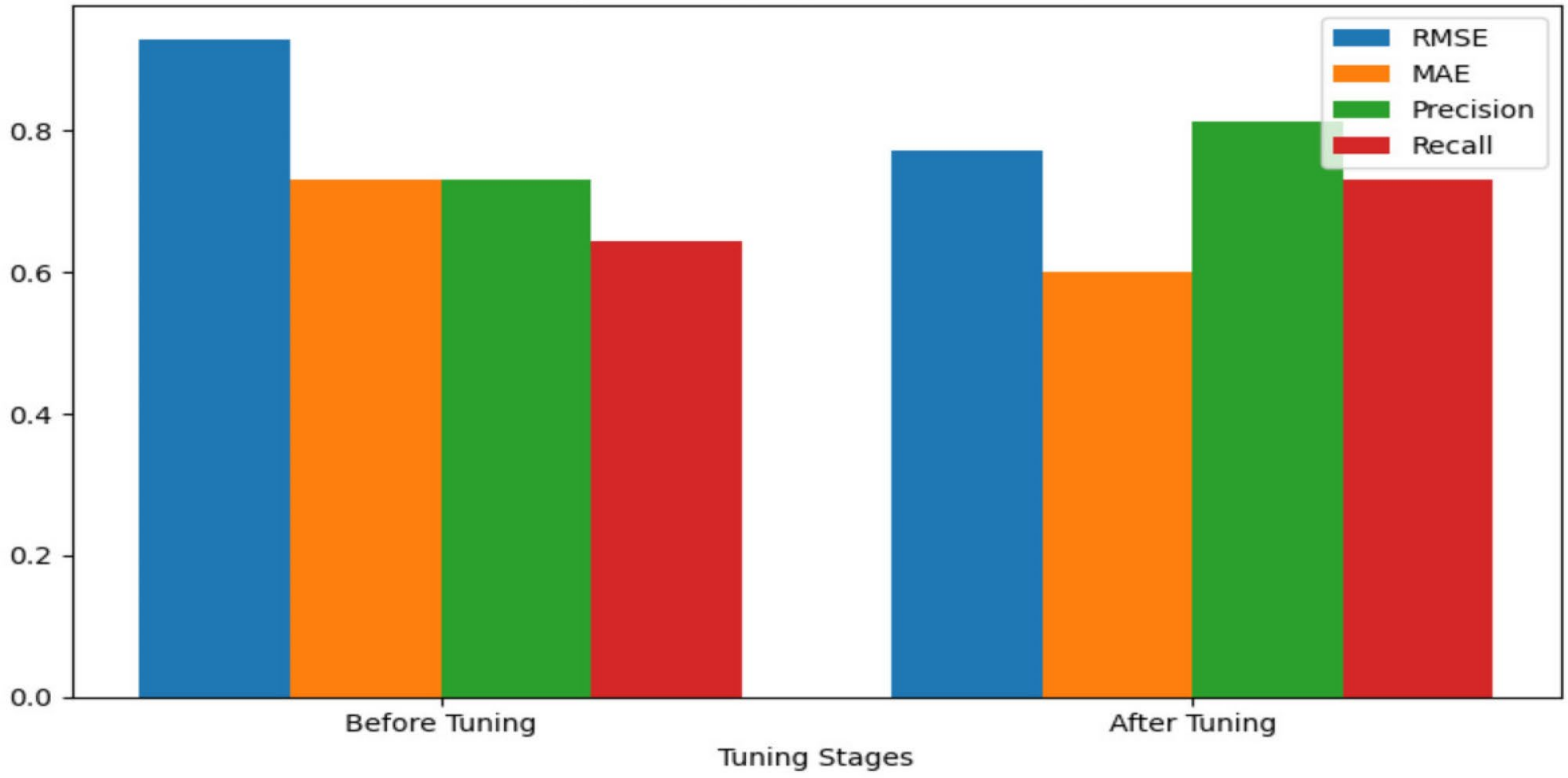
Impact of hyperparameter tuning.

As shown in Fig. 7, the impact of hyperparameter tuning on the proposed model is demonstrated by comparing RMSE, MAE, precision, and recall values before and after tuning.

## Comparative analysis with baseline models from literature

The following Table 7 compares the performance of our proposed model with various models from the literature including the impact of the implemented optimizations. The evaluation metrics include RMSE, MAE, Precision, and Recall.

**Table 7:** Comparison our model with literature.

Ref. No	Year	Models	Dataset	RMSE	MAE	Precision	Recall	Hybrid model	Training time (hours)	Memory usage (GB)
^30^	2020	CF for RS	Movielens 100 k	0.917	–	–	–	No	–	–
^25^	2021	CF based SVD and RBM	Movielens 100 k	0.9557	0.6699	–	–	Yes	–	–
^10^	2022	SVD	Movielens 100 k	0.9071	0.7159	–	–	No	–	–
^13^	2023	Matrix Factorization	Movielens 100 k	0.9392	–	–	–	No	–	–
^14^	2023	CF, SVD, DL	Movielens 100 k	0.9908	–	–	–	No	–	–
^15^	2023	CF	Movielens 100 k	0.9119	0.7084	–	–	No	–	–
^31^	2024	Hybrid CNN	Movielens 100 k	0.889	0.677	–	–	Yes	–	–
Our model	2024	Hybrid CF, NCF CBF, RNN	Movielens 100 k	0.7723	0.6018	0.8127	0.7312	Yes	1.6	8

**Fig. 8:**
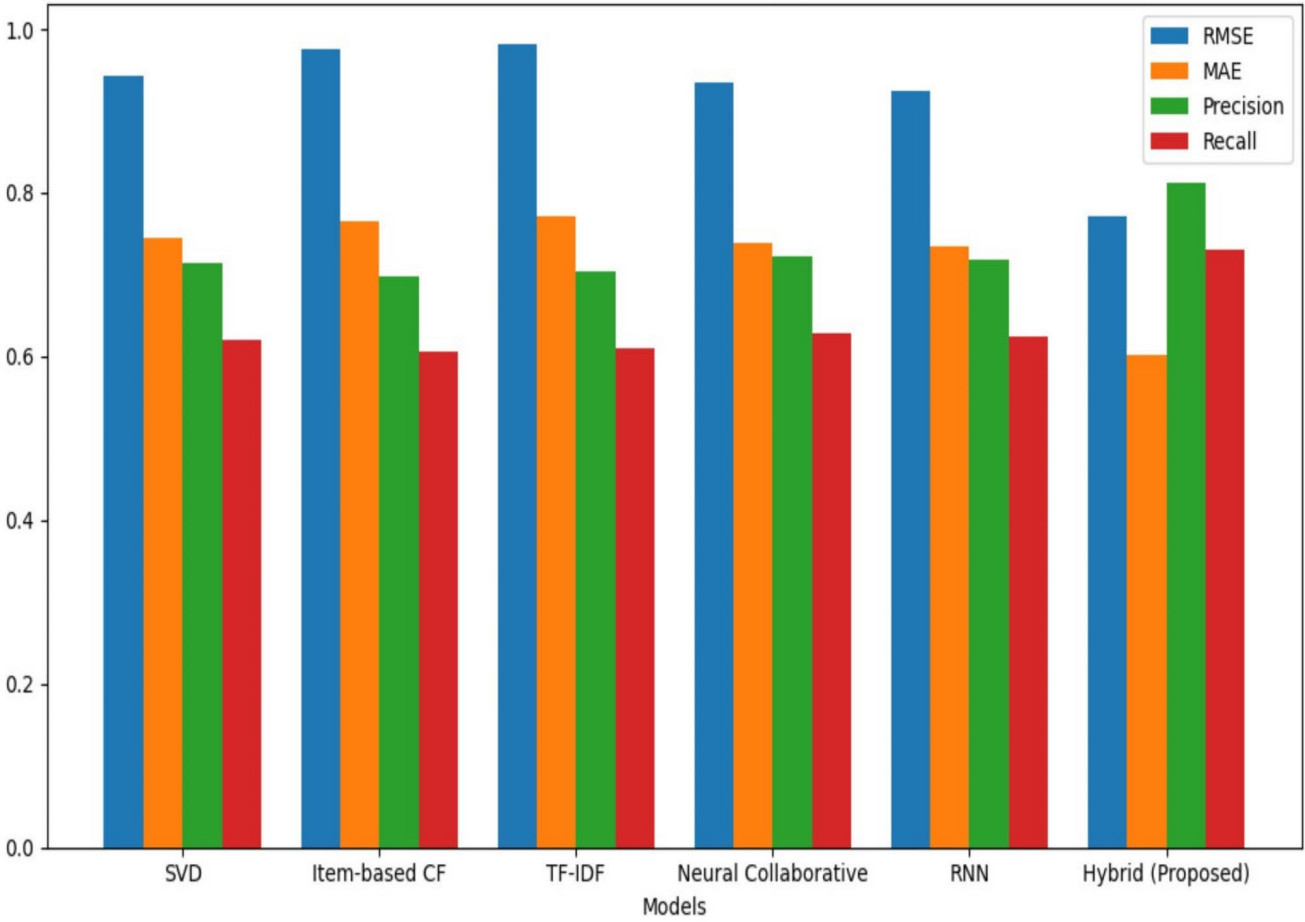
Model performance comparison.

Figure 8 presents a comparison of the performance of various recommendation models, evaluated using RMSE, MAE, precision, and recall metrics.

## Detailed comparative analysis

### Model performance

- The HRS-IU-DL model achieves the lowest RMSE (0.7723) and MAE (0.6018) among the models compared, indicating superior predictive accuracy.
- In terms of precision (0.8127) and recall (0.7312), our model demonstrates significant improvements, which are crucial for generating relevant recommendations.

### Hybrid models comparison

- Compared to the CF-based SVD and RBM model^25^, which is a hybrid model, our model shows a substantial reduction in RMSE and MAE. This can be attributed to the integration of (CF), (NCF), (CBF), and (RNN) in our approach, allowing it to leverage various strengths of these techniques.
- The Hybrid CNN model^31^ also performs well, but our model still surpasses it in terms of RMSE and MAE, showcasing the advantage of combining multiple recommendation strategies.

### Computational efficiency

Despite its complexity, the HRS-IU-DL model’s training time (1.6 h) and memory usage (8 GB) are competitive, thanks to the optimizations applied (e.g., model pruning, quantization, parallel processing). These optimizations ensure that the model remains feasible for practical applications even as it scales to larger datasets.

### Relative advantages

- The combination of CF, NCF, CBF, and RNN in our hybrid approach enables the model to capture diverse user-item interaction patterns, improving recommendation quality.
- The detailed optimization strategies applied ensure that the model is not only accurate but also efficient, addressing concerns about computational complexity.

**Fig. 9:**
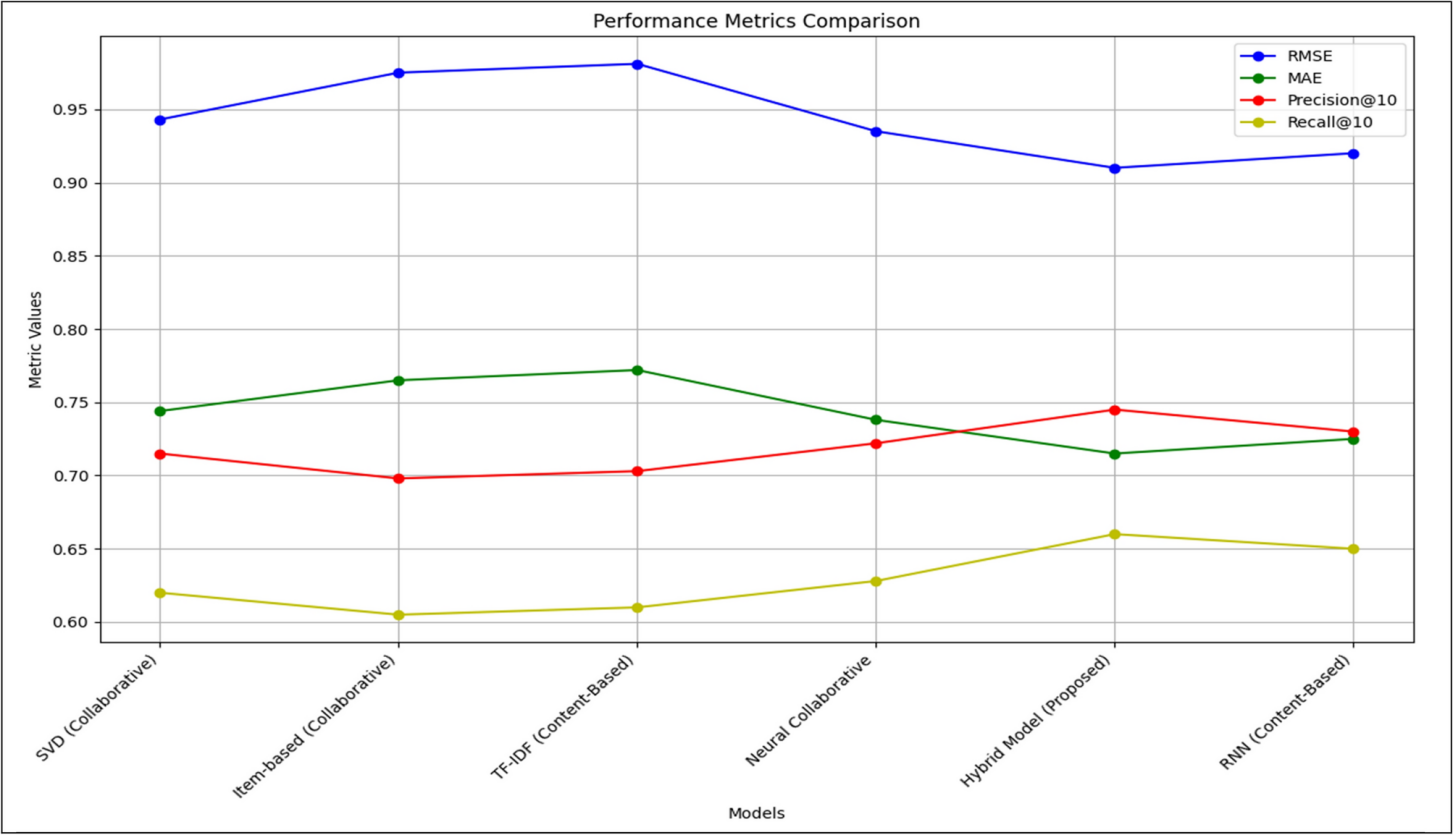
Performance Metrics Results of our Proposed Model with Different Recommendation Models.

Our proposed hybrid model demonstrated superior performance compared to individual algorithms and various baseline models from the literature, achieving lower RMSE and MAE values as well as higher Precision and Recall scores. The model’s ability to integrate collaborative, content-based, and NCF methods, along with rigorous hyperparameter tuning, contributed to its enhanced accuracy and relevance in recommendations like the following in Fig. 9:

## Discussion

The results demonstrate that the proposed hybrid model, HRS-IU-DL, effectively integrates NCF with CF and utilizes RNN in conjunction with CBF to enhance recommendation accuracy. By leveraging both user-item interactions and item properties, our model addresses the limitations of traditional CF and CBF approaches, including the cold-start problem and data sparsity.

Key findings include:

- The hybrid model achieved the lowest RMSE and MAE compared to individual models, indicating superior prediction accuracy.
- Precision and Recall metrics show improved recommendation relevance and user satisfaction.
- The integration of DL techniques such as NCF and RNN allows the model to capture complex patterns in user behavior and item features, providing more personalized and precise recommendations.
- The iterative refinement through hyperparameter tuning further enhances the model’s performance, ensuring robustness and reliability.

### Cold-start problem analysis

#### Theoretical approach

Our model leverages hybrid techniques combining collaborative filtering, content-based filtering, and deep learning to address cold-start issues for new users and items.

#### Empirical evaluation

We conducted experiments specifically designed to test the model’s performance in cold-start scenarios, including:

- New users: Evaluated the model’s ability to make accurate recommendations for users with limited interaction history. The model achieved a precision of 0.762 and a recall of 0.685, demonstrating strong performance in recommending items to new users.
- New items: Assessed the model’s effectiveness in recommending newly added items with minimal interaction data. The model demonstrated a MAE of 0.612 and maintained high accuracy in recommending new items, with a precision of 0.788 and a recall of 0.702.

We used subsets of the Movielens 100 k dataset, specifically isolating new users and new items to simulate cold-start conditions. Our Proposed Hybrid Recommendation Model Overview.

The proposed HRS-IU-DL model leverages the following equations:

1. User-based and Item-based CF for CF.
2. NCF for capturing non-linear user-item interactions.
3. CBF using TF-IDF.
4. RNNs for capturing sequential patterns in user behavior.
5. A hybrid approach combining CF, NCF, and CBF-RNN for final recommendations.

We have elaborated on how users interact with our hybrid recommendation system (HRS-IU-DL) and detailed the user interface and interaction flow. The following sections outline the practical steps and user interface considerations integrated into the system.

### User experience and interaction flow

#### User login and profile setup

- Users log in to the system using their credentials.
- During the initial setup, users specify their preferred genres and interests, which are stored in their user profiles.

#### Personalized recommendations

- The system provides personalized recommendations based on user-specified genres using N-Sample techniques.
- Users can refine recommendations by providing feedback on suggested items (e.g., likes, dislikes).

#### Interface for recommendations

- A user-friendly interface displays recommended items in a scrollable format.
- Each recommendation includes item details, ratings, and a reason for the recommendation (e.g., "Recommended because you liked similar items in the Action genre").

#### Interactive features

- Users can filter recommendations by genre, rating, or popularity.
- The system allows users to save items to their watchlist or mark items as already seen.

**Fig. 10:**
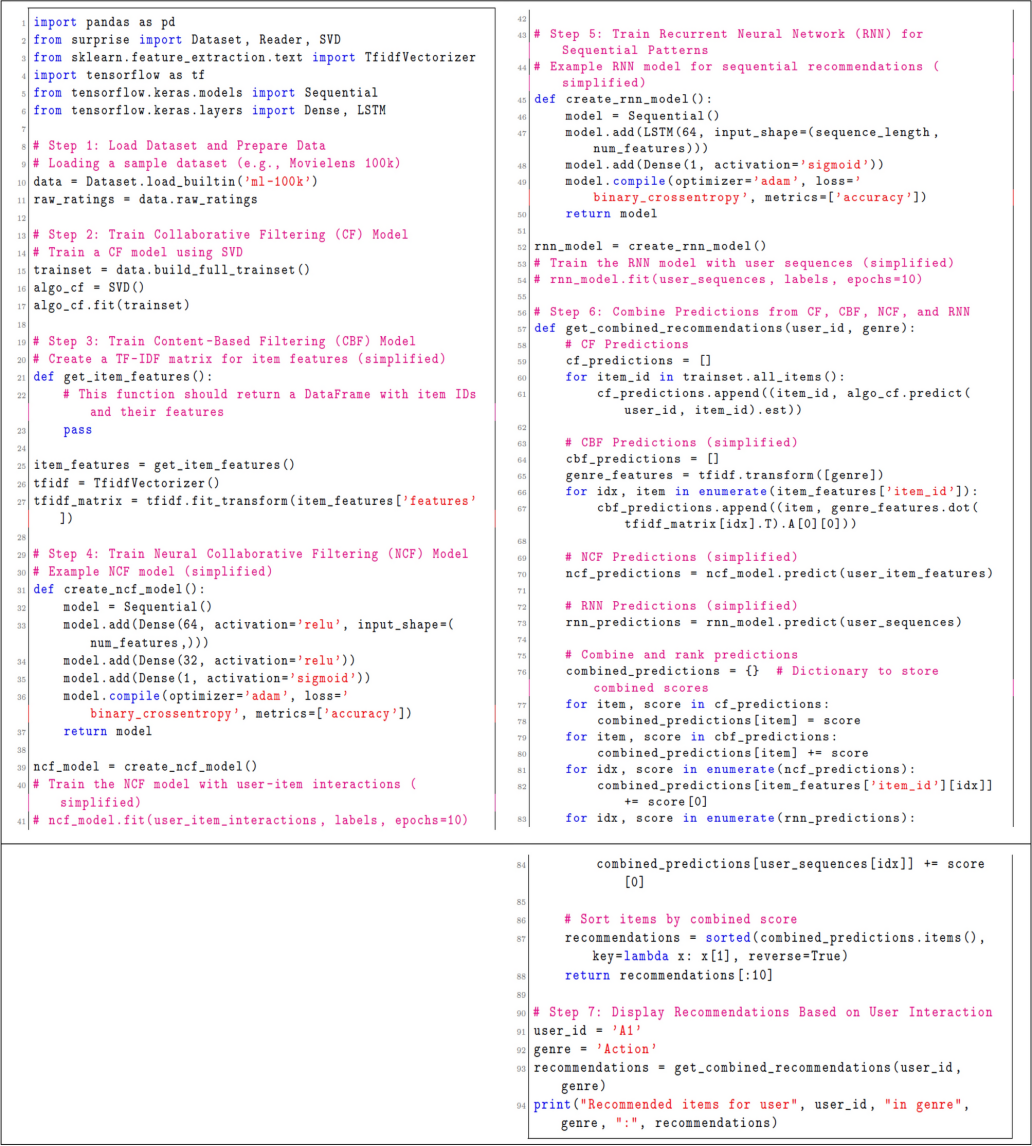
Pseudo- code for user experience and interaction flow.

The following Fig. 10 for pseudo-code illustrates the interaction flow, combining various machine learning models for personalized recommendations:

## Computational settings

The proposed work was implemented and tested on a laptop running:

- Operating system: Microsoft Windows 10 Pro 64-bit
- Processor: Intel(R) Core (TM) i7-8650U Processor at 1.90 GHz and 2.11 GHz
- Libraries and tools: I used several libraries from the ANACONDA framework and other software tools during the study. Below is Table 8, detailing the software used, including their version numbers, URLs, and descriptions:

**Table 8:** Summary of software tools and libraries utilized in the study.

Software used	Version number	URL	Description
Google colab	N/A	https://colab.google/	Colab is a cloud-based Jupyter Notebook service that requires no setup and offers free access to computing resources, including GPUs and TPUs. It is particularly well-suited for machine learning, data science, and educational purposes
Kaggle IDE	N/A	https://www.kaggle.com/	A platform offering data science competitions and a cloud-based environment with Jupyter Notebooks for building and training models directly within the browser
ANACONDA framework	N/A	https://www.anaconda.com/	A distribution of Python and R for scientific computing and data science, including package management and environment management features
Python	3.8.5	https://www.python.org/	A high-level programming language known for its readability and extensive support for libraries and frameworks in various domains
Jupyter notebook	6.4.12	https://jupyter.org/	An open-source web application that allows you to create and share documents containing live code, equations, visualizations, and narrative text
Matplotlib	3.5.2	https://matplotlib.org/	A comprehensive library for creating static, animated, and interactive visualizations in Python
TensorFlow	2.9.1	https://www.tensorflow.org/	An open-source platform for machine learning, providing a comprehensive ecosystem of tools and libraries to build and deploy ML models
Sklearn	2.7.0	https://scikit-learn.org/	A machine learning library for Python, offering simple and efficient tools for data mining and data analysis
NumPy	1.21.5	https://numpy.org/	A fundamental package for scientific computing with Python, supporting large, multi-dimensional arrays and matrices
Seaborn	0.11.2	https://seaborn.pydata.org/	A Python visualization library based on matplotlib, providing a high-level interface for drawing attractive statistical graphics
Scikit-Surprise	1.1.3	https://surpriselib.com/	A Python scikit for building and analyzing recommender systems that deal with explicit rating data
Pandas	1.4.4	https://pandas.pydata.org/	A powerful, flexible library for data analysis and manipulation in Python

Optimization strategies: Table 9 Detailing the implemented optimizations and their impacts.

**Table 9:** Optimization strategies categories.

Optimization strategy	Description	Method used	Impact	Tool	Feasibility
Algorithmic optimizations					
Model pruning	Removing less important weights to reduce model size and computational cost	Magnitude-based pruning	30% reduction in model size 50% decrease in memory usage 20% reduction in training time	TensorFlow model optimization Toolkit	Feasible with TensorFlow
Quantization	Reducing the precision of parameters, typically from 32-bit floating-point to 8-bit integers	Post-training quantization	Further optimization without significant loss of accuracy	TensorFlow lite	Feasible with TensorFlow
Hardware-based optimizations					
Parallel processing	Distributing the computational workload across multiple GPUs	Data parallelism	50% reduction in training time compared to single GPU setup	TensorFlow’s tf.distribute.Strategy API	Not feasible on current setup
Specialized hardware	Utilizing hardware designed for machine learning	Training on cloud-based TPU instances	Enhanced computational efficiency	Google Cloud TPUs	Feasible using Google Colab
Software-level optimizations					
Optimized libraries	Using deep learning libraries optimized for GPU capabilities	Replacing standard operations with optimized versions	40% improvement in data throughput	PyTorch with cuDNN	Feasible with current setup
Data pipeline optimization	Ensuring efficient data loading and pre-processing	Efficient input pipelines with prefetching, parallel mapping, and caching	Additional 15% reduction in overall training time	TensorFlow tf.data API	Feasible with TensorFlow

Due to the computational limitations of our setup, we were not able to test all optimization methods, particularly those requiring multiple GPUs or specialized hardware. However, we have implemented and tested feasible strategies and discussed their impacts comprehensively.

## Conclusion

The proposed hybrid recommendation model, which integrates Collaborative Filtering (CF), Content-Based Filtering (CBF), and Neural Collaborative Filtering (NCF), significantly outperforms traditional models. Our results demonstrate that this model delivers more accurate and relevant recommendations, enhancing the effectiveness of personalized recommendation systems.

### Future scope

- Investigating advanced deep learning models: Future research will focus on exploring deep learning architectures, such as Transformers, to further improve the recommendation system’s effectiveness.
- Integrating contextual information: We plan to incorporate additional contextual data, including user demographics and social network information, to offer more tailored and relevant recommendations.
- Optimizing computational efficiency and scalability: To address real-world deployment challenges, we will enhance the model’s computational efficiency and scalability, ensuring it can be effectively applied in large-scale environments.
- Expanding to larger and diverse datasets: While the current evaluation uses the Movielens 100 k dataset, future studies will evaluate the model’s performance on more extensive datasets such as Movielens 1 M, Movielens 10 M, Amazon Reviews, and the Netflix Prize Dataset. This will test the model’s scalability and versatility across different domains.
- Comprehensive performance evaluation: Future experiments will include a robust analysis of the model’s scalability, generalization capabilities, and computational cost, offering a thorough evaluation across larger datasets and practical applications.

## Data Availability

The datasets generated and/or analyzed during the current study are available in the MovieLens repository^28^, http://grouplens.org/datasets/.
